# Efficacy and Safety of Ibuprofen for Pain Resolution After Laparoscopic Cholecystectomy: A Systematic Review and Meta-Analysis

**DOI:** 10.7759/cureus.104690

**Published:** 2026-03-04

**Authors:** Adib Siddiki, Salah Ahmed, Zurnish Rauf, Soban Haider, Zainab Khalid, Muhammad Ali Sumbal, Shinza Chishti, Minahil Faheem, Olumide A Mogbojuri, Zeeshan Alvi

**Affiliations:** 1 General Surgery, Holy Family Red Crescent Medical College and Hospital, Dhaka, BGD; 2 Medicine and Surgery, University of Nairobi, Nairobi, KEN; 3 Medicine and Surgery, Jinnah Hospital, Lahore, PAK; 4 Orthopedics and Trauma, Islamic International Medical College Trust (IIMCT) Pakistan Railways Hospital, Islamabad, PAK; 5 Medicine, CMH Institute of Medical Sciences (CIMS), Bahawalpur, PAK; 6 Medicine, Balfour Hospital, NHS, Kirkwall, GBR; 7 Pathology, Nishtar Medical University, Multan, PAK; 8 Surgery, Bakhtawar Amin Medical and Dental College, Multan, PAK; 9 General Medicine, University of Lagos, Lagos, NGA; 10 Public Health, York St John University, London, GBR

**Keywords:** intravenous ibuprofen, laparoscopic cholecystectomy, non-opioid analgesia, opioid consumption, post-operative pain

## Abstract

Pain control in the post-operative period is one of the key factors of patient satisfaction and post-operative recovery after laparoscopic cholecystectomy. The efficacy and tolerability of intravenous ibuprofen as a general analgesic in post-operative care following this procedure should be evaluated. A systematic review of randomized controlled trials was conducted in accordance with the Preferred Reporting Items for Systematic Reviews and Meta-Analyses (PRISMA) 2020 guidelines. The searches in PubMed, Embase, Scopus, and the Cochrane Library were performed through January 2026. In adults, patients undergoing laparoscopic cholecystectomy were compared between those receiving intravenous ibuprofen and those receiving placebo/control interventions. The primary endpoints considered post-operative pain, whereas opioid consumption and negative outcomes were the secondary endpoints. Random-effects models were applied. Five randomized controlled trials (RCTs) with 388 patients were included. Employing intravenous ibuprofen significantly reduced pain ratings at 1 h, 2 h, and 24 h after surgery. The ibuprofen group also had a significantly lower opioid consumption. The groups did not differ significantly in sedation rates. Nevertheless, ibuprofen was associated with a markedly lower rate of post-operative nausea and pruritus. There was a considerable heterogeneity in efficacy outcomes. Intravenous ibuprofen is a valuable and safe pain reliever following laparoscopic cholecystectomy and brings a great deal of pain reduction and opioid-sparing without augmenting adverse effects. Its inclusion in multimodal analgesic programs could be beneficial for postoperative recovery.

This study aimed to identify whether the intravenous administration of ibuprofen is effective for treating post-operative pain in patients having laparoscopic cholecystectomy. This study evaluates secondary outcomes using the following three different measurements: the need for extra pain medication, the complete recovery process, and the rate of all adverse effects. The objective of this research was to offer a comprehensive assessment of the use of ibuprofen in the prevention and treatment of post-laparoscopic cholecystectomy pain, and to give recommendations for information-based interventions that will enhance patient care with regard to surgical operations.

## Introduction and background

Laparoscopic cholecystectomy is broadly considered the gold standard in the treatment of symptomatic gallstone disease and is commonly used in surgery [1,2]. Although laparoscopic cholecystectomy is minimally invasive, patients often experience significant pain during the first 24-48 h, which can delay ambulation, extend hospitalization, and negatively impact recovery and patient satisfaction [3-5]. Pain management in the post-operative period is thus an essential aspect of the peri-operative care in laparoscopic cholecystectomy [3,6].

Post-operative pain after laparoscopic cholecystectomy is multifactorial, arising from incisional trauma, visceral manipulation, peritoneal irritation, and residual pneumoperitoneum [7]. Opioids are usually administered in order to help treat this pain, but are constrained by side effects like nausea, vomiting, sedation, respiratory depression, ileus, and dependency [8]. These restrictions have led to a greater interest in multimodal analgesic approaches that will reduce opioid use to the greatest extent possible and still effectively manage pain [9].

Non-steroidal anti-inflammatory drugs (NSAIDs) have become significant in the treatment of post-operative pain because of their analgesic, anti-inflammatory, and opioid-sparing effects [10]. Ibuprofen is a non-selective NSAID that inhibits cyclooxygenase activity, reducing prostaglandin production and thereby reducing inflammation and providing pain relief [11]. Multiple studies have demonstrated that ibuprofen effectively reduces post-operative pain and opioid consumption following laparoscopic cholecystectomy, but the overall evidence remains inconclusive regarding its use in this setting [10,12]. Thus, safety and effectiveness should be thoroughly assessed in the clinical setting. Considering the focus on opioid-sparing interventions and the optimal use of intravenous ibuprofen in patients undergoing laparoscopic cholecystectomy, this systematic review and meta-analysis would help justify the use of intravenous ibuprofen as an effective and safe pain management method and reduce adverse outcomes.

Rationale

Laparoscopic cholecystectomy is a common surgery, and with the post-operative pain, this is what defines the comfort of the patient, early ambulation, and patient recovery. The side effects of opioids, such as nausea, sedation, and the development of dependency, even though this might be viewed as evidence that they are the drugs that are employed to control the pain, lead to the adoption of safer and more effective drugs. NSAIDs, especially ibuprofen, can be used in analgesia and for anti-inflammatory purposes.

Ibuprofen is a simple and easily accessible analgesic and anti-inflammatory non-steroidal agent that can be used to manage post-operative pain. Evaluation of its efficacy and safety in this regard can provide a clear way to optimize pain management and improve recovery following laparoscopic cholecystectomy.

## Review

Study design and protocol

This systematic review and meta-analysis were conducted in accordance with the Preferred Reporting Items for Systematic Reviews and Meta-Analyses (PRISMA) 2020 guidelines [13]. To ensure methodological rigor and transparency, the study protocol was prospectively registered in PROSPERO (#CRD420261292724), where the objectives, eligibility criteria, and statistical approach were predefined. The methodological framework was based on the Cochrane Handbook for Systematic Reviews of Interventions (version 6.5) to maintain quality and minimize bias [14,15].

Data sources and search strategy

The researchers systematically searched the databases PubMed, Embase, Cochrane Library, and Scopus from the beginning of each database until January 2026, without language restrictions. The search strategy also used Medical Subject Headings (MeSH) and any other relevant free-text terms. Keywords employed in the intervention were ibuprofen, intravenous ibuprofen, and laparoscopic cholecystectomy (LC). The terms associated with the comparators were placebo and control. The study population was identified using the terms cholelithiasis, gallbladder disease, and cholecystectomy. Search terms related to the results, such as post-operative analgesic efficacy and safety, include pain score, visual analog scale (VAS), opioid consumption, nausea, sedation, and pruritus. Boolean operators (AND/OR) were employed to connect keywords and optimize search sensitivity. Besides searching electronic databases, reference lists of included studies, pertinent reviews, and previous meta-analyses were checked in the reference lists to find the eligible trials. The entire search approach used for each database is provided in the table in appendix.

Study selection

**Figure 1:**
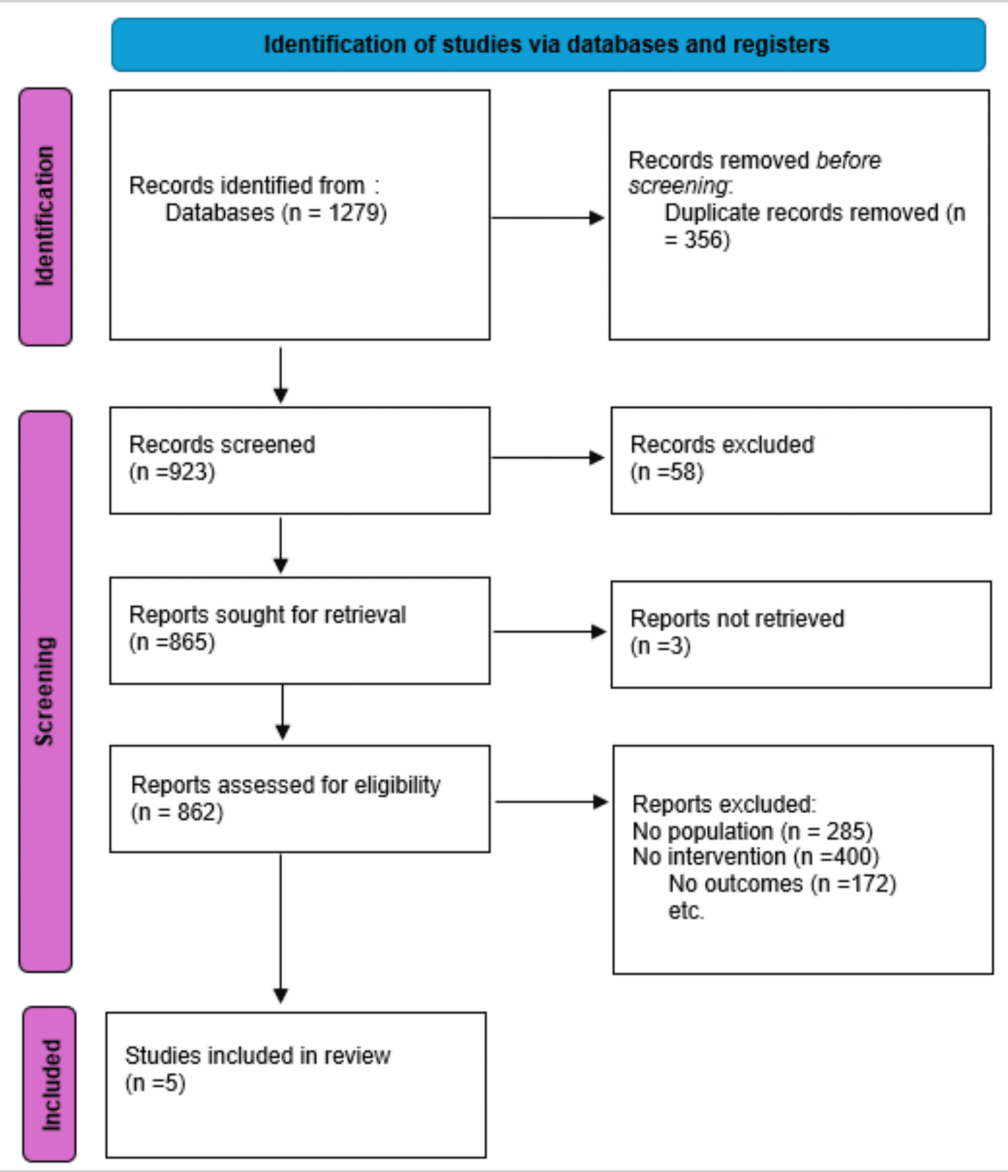
PRISMA flow diagram of included studies. This PRISMA 2020 flow diagram illustrates the process of study identification, screening, eligibility assessment, and final inclusion for the systematic review and meta-analysis evaluating the efficacy and safety of ibuprofen for post-operative pain resolution after laparoscopic cholecystectomy. Records were identified through database searching, followed by removal of duplicate records and screening of titles and abstracts. Full-text articles were subsequently assessed for eligibility, with exclusions based on predefined criteria including non-relevant population, outcomes, or interventions. A total of five studies met the inclusion criteria and were included in the final qualitative and quantitative synthesis. PRISMA: Preferred Reporting Items for Systematic Reviews and Meta-Analyses

The retrieved references were screened based on their titles, abstracts, and relevant outcomes, and assessed for eligibility according to predefined inclusion and exclusion criteria by two independent authors (Adib Siddiki and Salah Ahmed). Any discrepancies were resolved by a third independent reviewer (Zurnish Rauf). The conference presentations and grey literature were not included in the literature search. The study selection process, including identification, screening, eligibility assessment, and final inclusion, is illustrated in Figure 1.

Selection criteria

Selection criteria for study inclusion were structured using the Population, Intervention, Comparison, and Outcomes (PICO) framework. This review included adult patients aged ≥18 years who underwent laparoscopic cholecystectomy for benign gallbladder disease. Studies evaluating the use of intravenous ibuprofen as an intervention for post-operative pain control were considered eligible. Placebo or normal saline solution (usually used as a control) was one of the comparators of the study. The intensity of post-operative pain was considered by the researchers as the main outcome measure, and the researchers measured the intensity of post-operative pain using the validated tools of pain assessment, which include the visual analog scale and other equivalent methods of testing. Two secondary outcomes were measured by the researchers, and they included the evaluation of post-operative opioid use in terms of pain relief and the evaluation of safety outcomes associated with ibuprofen treatment, which consisted of nausea, sedation, and pruritus as side effects. The researchers did not include studies that employed non-randomized methods or studies with heterogeneous populations not aligned with the predefined eligibility criteria (e.g., participants with unrelated comorbid conditions or mixed disease etiologies without subgroup data) were excluded. Studies were eligible if they were randomized controlled trials evaluating the intervention of interest. Non-randomized studies, including cohort, case-control, cross-sectional, and single-arm interventional studies, as well as studies without a comparator group, were excluded. We also excluded case reports, narrative reviews, systematic reviews, editorials, letters to the editor, and conference abstracts that did not provide sufficient quantitative data for analysis. Studies were further excluded if they did not report measurable outcomes aligned with the predefined study objectives or if they contained duplicate or overlapping data from the same study population.

Screening process and study selection

Titles and abstracts were screened by two reviewers (Soban Haider and Zainab Khalid), who were independent to select preliminary eligible studies. The full-text articles were then evaluated using the set of inclusion criteria. The group agreed on something after their deliberations, and a third reviewer would be called to solve any outstanding differences.

Data extraction

A standardized form was used to extract data independently. The researchers retrieved data from the study using narrative extraction, comprising the first author, year of publication, country, study design, and sample size. The researchers recorded the demographic details of the patients, such as age and sex distribution, American Society of Anesthesiologists (ASA) physical status, and body mass index as soon as they were available. Researchers collected data on the intervention, including the ibuprofen dosage and route of administration, and the timing of administration relative to surgical operations. The researchers assessed the intensity of post-operative pain using the validated tools of pain measurement at the predetermined time intervals. The analgesic effectiveness measured by the researchers was based on the measurement of opioid consumption, which took place during the period of the patient's recovery in the aftermath of the surgery. The safety outcomes were evaluated by the team of researchers, who monitored adverse events such as nausea, sedation, and pruritus. The researchers employed a descriptive data synthesis technique to develop an overall summary of the efficacy and safety outcomes from other studies. Any discrepancies during data extraction were resolved by consensus, with arbitration by a third reviewer (Muhammad Ali Sumbal) when required (Table 1).

**Table 1:** Baseline characteristics and demography of included studies. Note: values are presented as mean±SD or number of patients, as appropriate. The control group received placebo or standard care as specified in individual studies. M/F: male/female; ASA: American Society of Anesthesiologists; NR: not reported

Studies	Location	Group	n	Age (years, mean±SD)	Gender (M/F)	ASA (I/II)	BMI (mean±SD)	Drug and dose
Erdi et al. (2022) [16]	Ardabil, Iran	Ibuprofen	30	43.53±10.13	8/22	NR	NR	Ibuprofen 800 mg IV
		Placebo (control)	30	43.56±11.51	7/23	NR	NR	Normal saline IV
Ekinci et al. (2020) [17]	Istanbul, Turkey	Ibuprofen	30	43.06±7.88	13/17	11/19	26.00±1.92	Ibuprofen 800 mg IV
		Placebo (control)	30	44.06±8.32	15/15	18/12	26.53±1.54	100 mL saline IV
Soyalp et al. (2025) [18]	Van and Erzurum, Turkey	Ibuprofen	30	45.47±11.32	13/17	12/18	28.20±3.20	Ibuprofen 800 mg IV
		Placebo (control)	30	45.70±12.07	15/15	14/16	28.90±4.45	100 mL 0.9% NaCl IV
Karaca et al. (2017) [19]	Konya, Turkey	Ibuprofen+pregabalin	29	46.76±10.69	18/11	19/10	28.17±4.06	Ibuprofen 400 mg IV+pregabalin 150 mg oral
		Pregabalin (control)	29	43.66±11.10	15/14	20/9	26.85±2.12	Pregabalin 150 mg oral+IV saline
Ahiskalioglu et al. (2017) [15]	Erzurum, Turkey	Ibuprofen	30	50.60±10.27	17/13	16/14	164.93±8.20	400 mg IV ibuprofen
		Control	30	46.93±12.37	19/11	10/20	168.40±6.25	Placebo (saline)

Risk of bias assessment

The methodological quality of the included randomized controlled trials was independently assessed by two reviewers (Shinza Chishti and Minahil Faheem) using the Cochrane risk of bias tool (RoB 2.0). Disagreements were resolved through discussion or by third-party (Zeeshan Alvi) adjudication. Based on the maximum risk level in each domain, an overall risk-of-bias judgment was made for each study. Two reviewers independently completed the assessment, and any discrepancies were resolved through discussion until agreement was reached. A traffic-light diagram was used to visually summarize the quality assessment results.

Data analysis

Statistical analyses were conducted using Review Manager (RevMan) version 5.4 (London, UK: The Cochrane Collaboration). Continuous outcomes (pain scores and opioid consumption) were pooled using the inverse-variance random-effects model and reported as mean differences (MDs) with 95% confidence intervals (CIs). Dichotomous outcomes (nausea, sedation, and pruritus) were analyzed using the Mantel-Haenszel random-effects model and expressed as risk ratios (RRs) with 95% CIs. Statistical heterogeneity was assessed using the chi-square test and quantified using the I² statistic; values >50% indicate substantial heterogeneity. A p<0.05 was considered statistically significant.

Results

Baseline Characteristics and Patients’ Demography

A total of 388 patients undergoing laparoscopic cholecystectomy were included in the five randomized controlled trials that were published between 2017 and 2025. Each study had a sample size of 58-60, and they were all carried out in Iran and Turkey. Patients were distributed equally between the ibuprofen and comparator groups in every trial. Most of the patients were in their fourth or sixth decade of life. Comparator groups showed similar age distributions, ranging from 36.16±9.67 to 45.70±12.07 years, while ibuprofen-treated groups had mean ages ranging from 43.06±7.88 to 50.60±10.27 years. Within individual studies, there were no age differences between treatment arms that were clinically significant. The distribution of genders varied among research, but most cohorts showed a female predominance, which is consistent with the higher incidence of gallstone disease in women. Across all included studies, the percentage of male participants ranged from 27% to 57%.

Based on the ASA physical status classification, most patients were categorized as low perioperative risk. ASA class I or II was allocated to most patients, and the distribution between the ibuprofen and control groups was similar. When available, mean ASA classifications showed that the surgical population was generally in good health. The majority of patients were overweight, according to three trials that provided body mass index (BMI), with mean values ranging from 26.00±1.92 to 28.90±4.45 across treatment arms. Within individual trials, there were no discernible differences in baseline BMI between the intervention and comparator groups. In terms of analgesic treatments, 400 mg or 800 mg of intravenous ibuprofen was given either alone or in conjunction with pregabalin. Comparator groups were given either active analgesics, such as acetaminophen, dexketoprofen, or pregabalin, or a placebo (normal saline). The risk of confounding due to baseline imbalance was reduced since baseline clinical and demographic parameters were generally well matched among research groups in Table 1.

Meta-analysis of outcomes

Efficacy Outcomes

**Figure 2:**
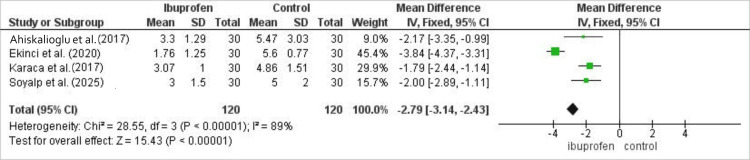
Meta-analysis of post-operative pain scores at 1 h following laparoscopic cholecystectomy comparing intravenous ibuprofen with control. Data are presented as mean difference with 95% confidence intervals. Negative values favor ibuprofen. Heterogeneity was assessed using the I² statistic.

In the pooled analysis of 240 patients (120 ibuprofen, 120 control), ibuprofen significantly reduced visual analog scale pain scores at 1 h post-operatively, with a mean difference of -2.64 (95% CI: -2.99 to -2.30; p<0.00001), though considerable heterogeneity was present (I²=89%). The pooled analysis of post-operative pain scores at 1 h is shown in Figure 2.

**Figure 3:**
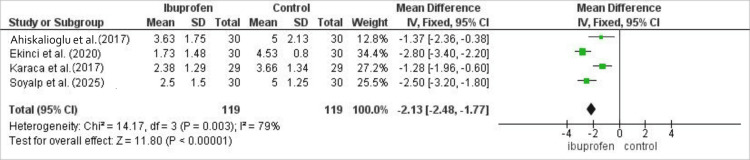
Meta-analysis of post-operative pain scores at 2 h following laparoscopic cholecystectomy comparing intravenous ibuprofen with control. Data are presented as mean difference with 95% confidence intervals. Negative values favor ibuprofen. Heterogeneity was assessed using the I² statistic.

Among 238 patients (119 per group), ibuprofen continued to provide superior analgesia at 2 h, yielding a mean difference of -2.13 (95% CI: -2.48 to -1.77; p<0.00001), with substantial heterogeneity across studies (I²=79%). The forest plot summarizing post-operative pain scores at 2 h is shown in Figure 3.

**Figure 4:**
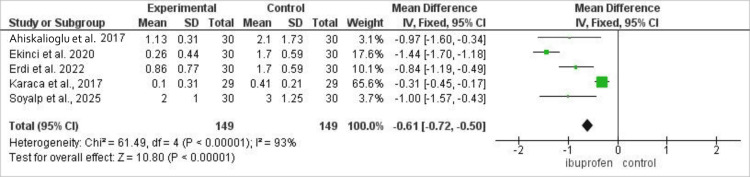
Forest plot comparing post-operative pain scores between intravenous ibuprofen and control after laparoscopic cholecystectomy. Effect estimates are presented as mean differences with 95% confidence intervals. Negative values favor ibuprofen. Statistical heterogeneity was assessed using the I² statistic.

Analysis of 298 patients (149 ibuprofen, 149 control) showed that ibuprofen was associated with significantly lower odds of higher pain scores at 24 h, with a mean difference (MD) of -0.61 (95% CI: -0.72 to -0.51, p<0.00001) with high heterogeneity (I^2^=93%). The comparison of post-operative pain scores at 24 h between the ibuprofen and control groups is illustrated in Figure 4.

**Figure 5:**
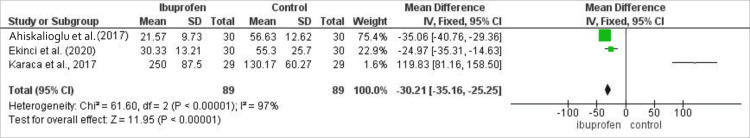
Forest plot comparing post-operative opioid consumption between intravenous ibuprofen and control following laparoscopic cholecystectomy. Effect estimates are presented as mean differences with 95% confidence intervals. Negative values indicate reduced opioid consumption in the ibuprofen group. Statistical heterogeneity was assessed using the I² statistic.

In 178 patients (89 per group), ibuprofen significantly reduced post-operative opioid consumption by a mean of 30.21 units (95% CI: -35.16 to -25.25; p<0.00001), despite very high heterogeneity among the included studies (I²=97%). The effect of intravenous ibuprofen on post-operative opioid consumption is summarized in Figure 5.

Safety Outcomes

**Figure 6:**
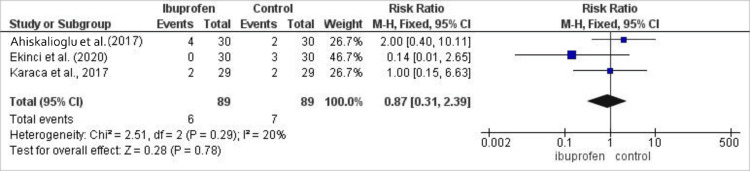
Forest plot comparing the incidence of post-operative sedation between intravenous ibuprofen and control following laparoscopic cholecystectomy. Effect estimates are presented as risk ratios with 95% confidence intervals. Values <1 favor ibuprofen. Statistical heterogeneity was assessed using the I² statistic.

**Figure 7:**
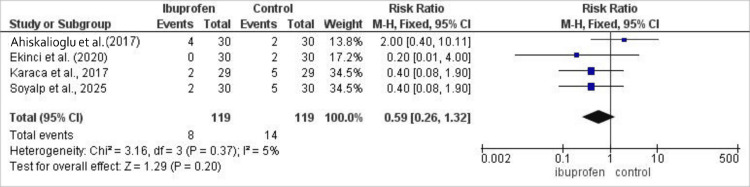
Forest plot comparing the incidence of post-operative pruritus between intravenous ibuprofen and control following laparoscopic cholecystectomy. Effect estimates are presented as risk ratios with 95% confidence intervals. Values <1 favor ibuprofen. Statistical heterogeneity was assessed using the I² statistic.

Evaluation of sedation in 178 patients (89 on ibuprofen, 89 controls) revealed no significant difference between groups (risk ratio: 0.87; 95% CI: 0.31-2.39; p=0.78), with low heterogeneity (I²=20%). The comparative incidence of post-operative sedation between treatment groups is presented in Figure 6. Among 238 patients (119 per group) in four studies, ibuprofen significantly reduced the incidence of pruritus (odds ratio: 0.36; 95% CI: 0.19-0.69; p=0.002), and there was no heterogeneity across studies (I²=5%) (Figure 7) [15,17-19].

**Figure 8:**
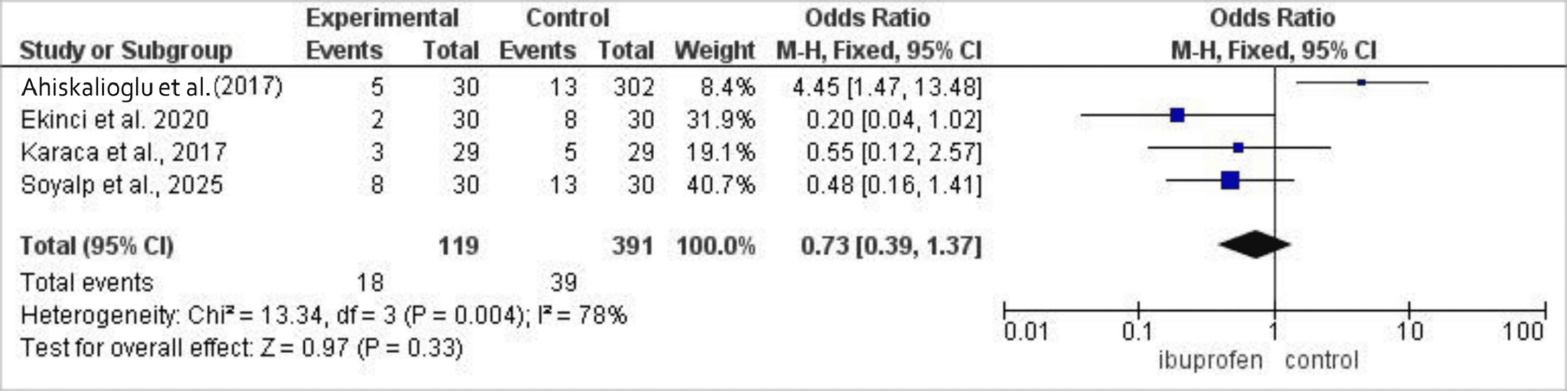
Forest plot comparing the incidence of post-operative nausea between intravenous ibuprofen and control following laparoscopic cholecystectomy. Effect estimates are presented as odds ratios with 95% confidence intervals using a random-effects model. Values <1 favor ibuprofen. Statistical heterogeneity was assessed using the I² statistic.

In the same cohort of 246 patients across five studies, ibuprofen was also associated with a significant reduction in post-operative nausea, demonstrating an odds ratio of 0.36 (95% CI: 0.19-0.69; p=0.002) and consistent effects across trials (I²=78%) (Figure 8) [15,17-19].

Quality Assessment

**Figure 9:**
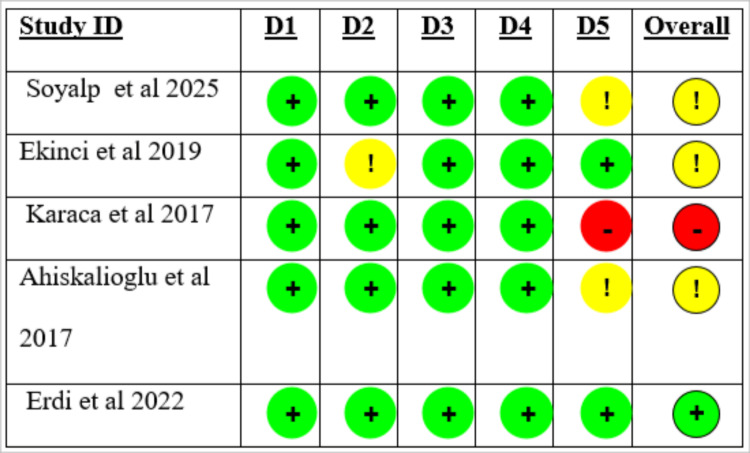
Risk of bias assessment of included studies evaluating ibuprofen for post-operative pain after laparoscopic cholecystectomy. Risk of bias was assessed using the Cochrane risk of bias 2.0 tool. Green circles indicate low risk of bias, yellow circles indicate some concerns, and red circles indicate high risk of bias across individual domains. D1 represents bias arising from the randomization process; D2 represents bias due to deviations from intended interventions; D3 represents bias due to missing outcome data; D4 represents bias in measurement of the outcome; and D5 represents bias in selection of the reported result. The overall risk of bias for each study reflects the highest risk identified in any domain.

**Figure 10:**
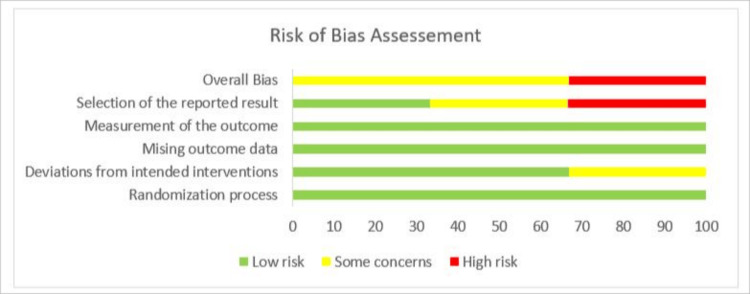
Traffic plots of risk of bias across included studies. Risk of bias was assessed using the Cochrane risk of bias tool. Green indicates low risk, yellow indicates some concerns, and red indicates high risk of bias across domains.

The risk of bias assessment includes five randomized controlled studies. Overall, one study was deemed to have a low risk of bias, three had some issues, and one had a high risk of bias. The survey by Erdi et al. demonstrated rigorous methodology, with a low risk of bias across all five domains [16]. On the other hand, Ahiskalioglu et al., Ekinci et al., and Soyalp et al. raised some general concerns, primarily due to selective outcome reporting [15,17,18]. Overall, Karaca et al. were found to have a high risk of bias, primarily due to a high risk in the selection domain of the stated results [19]. For the randomization procedure, missing outcome data, and outcome measurement, all included studies were deemed to be at low risk of bias across the different domains, with appropriate randomization, low attrition, and trustworthy outcome assessment. Due to inadequate reporting, one study's deviations from planned interventions prompted some concerns. The reported result was chosen as the domain most commonly linked to bias; in several studies, there was insufficient evidence of outcome prespecification, which could have affected the validity of the reported results. The domain-wise and overall risk of bias across included studies is presented in Figures 9, 10.

Discussion

The study used meta-analysis and systematic review methods to evaluate the effects of intravenous ibuprofen on surgical outcomes after laparoscopic cholecystectomy. The high heterogeneity observed across efficacy outcomes (I²=79-97%) likely reflects differences in ibuprofen dosing, timing of administration, comparator analgesics, anesthesia protocols, and study populations. Despite this variance, the direction of effect was consistently in favor of intravenous ibuprofen, which supports the soundness of the results.

Our analysis showed that ibuprofen treatment reduced early post-operative pain scores at both the 1- and 2-h time points compared with the control group. These findings are consistent with prior randomized controlled trials reporting reduced VAS pain scores at rest and during activity, along with decreased post-operative opioid requirements in patients receiving intravenous ibuprofen [20]. Our analysis further demonstrated that intravenous ibuprofen reduced 24-h post-operative pain. This finding is in line with a recent systematic review and meta-analysis that evaluated 23 randomized controlled trials and reported lower VAS scores at 24 h with intravenous ibuprofen compared with placebo, along with modest but clinically meaningful relief of late post-operative pain [21]. By focusing specifically on laparoscopic cholecystectomy, the present meta-analysis adds procedure-specific evidence supporting the sustained analgesic benefit of intravenous ibuprofen.

These findings are further enhanced by recent evidence. A meta-analysis of multimodal analgesic approaches involving the use of NSAIDs showed that these interventions are related to better post-operative pain management and less opioid use after laparoscopic cholecystectomy [6]. Also, a new randomized controlled trial indicated that intravenous ibuprofen has the same or even better analgesic effects compared to acetaminophen, with a decrease in opioid needs [12]. These newer studies favor the incorporation of intravenous ibuprofen into the existing multimodal analgesic regimens.

Ibuprofen in our analysis showed a significant reduction in post-operative opioid use over control. This observation is consistent with previous systematic reviews and randomized controlled trials, which reported that intravenous ibuprofen markedly reduces cumulative opioid requirements up to 24 h after surgery [22]. Reduced opioid exposure may contribute to improved post-operative recovery and patient comfort. The decreased exposure to opioids is likely to lead to better post-operative outcomes and patient comfort. In our analysis, ibuprofen did not show a significant risk of sedation compared to the control. This finding aligns with recent randomized trials, which have shown no increased risk of adverse events and that intravenous ibuprofen is well tolerated [23].

Furthermore, ibuprofen was associated with a significant reduction in post-operative nausea and pruritus. This reduction is most likely attributable to decreased opioid consumption rather than a direct pharmacological antiemetic effect of ibuprofen, as opioid-related adverse effects are known to be dose-dependent [24].

Most of the selected studies had satisfactory methodological rigor, and only one trial was rated as having a high risk of bias. Despite certain problems with selective reporting, the overall uniformity of the results indicates that these constraints do not have a serious chance of diminishing the conclusions greatly. Importantly, these findings are also consistent with recent Procedure-Specific Postoperative Pain Management (PROSPECT) guideline recommendations, which advocate the use of NSAIDs as a key component of multimodal, opioid-sparing analgesic regimens following laparoscopic cholecystectomy [25]. Collectively, these findings warrant the incorporation of intravenous ibuprofen in multimodal, opioid-sparing analgesic regimens, better post-operative outcomes, decreased opioid side effects, and increased patient satisfaction.

Limitations

There are a few limitations that need to be mentioned. Initially, the number of included studies and participants was small, which may limit the extrapolation of the results. Second, there was considerable heterogeneity in some efficacy outcomes, likely due to variation in ibuprofen dosing regimens, comparator drugs, and adjunct analgesic regimens. Third, the incorporated trials were all Iranian and Turkish, potentially limiting the generalizability of these findings to other healthcare settings and populations. Fourth, one of the studies included was at high risk of bias, and some studies had issues with selective reporting of outcomes, which may affect the pooled estimates. Lastly, the long-term effects (renal, gastrointestinal, or cardiovascular complications) were not adequately reported, preventing a complete evaluation of long-term safety.

Future directions

Future studies need to consider large, multicenter randomized controlled trials (RCTs) with standardized dosing, timing, and peri-operative analgesic regimens for ibuprofen to mitigate heterogeneity and enhance comparability. It would be beneficial to make direct comparisons of ibuprofen and other non-opioid analgesics in multimodal regimens. Also, the long-term safety outcomes, patient-reported recovery measures, and cost-efficacy should be evaluated in the studies. The assessment of ibuprofen as a component of standardized Enhanced Recovery After Surgery (ERAS) protocols can further elucidate its role in optimizing post-operative recovery following laparoscopic cholecystectomy.

## Conclusions

IV ibuprofen is a safe and effective analgesic in the treatment of post-operative pain in laparoscopic cholecystectomy. It results in a significant reduction of early and late pain scores, opioid consumption, and opioid-induced adverse events, including nausea and pruritus. These findings confirm its daily application in multimodal, opioid-sparing analgesic therapy to maximize recovery and patient satisfaction.

## Appendices

**Table 2:** Search approach used for each database. PubMed:320; Embase:831: (('ibuprofen'/exp OR ibuprofen OR 'non steroidal anti inflammatory drug'/exp OR NSAID) AND ('laparoscopic cholecystectomy'/exp OR 'laparoscopic cholecystectomy' OR 'laparoscopic gallbladder removal') AND ('postoperative pain'/exp OR 'postoperative pain' OR analgesia OR pain); Scopus:124: (laparoscopic cholecystectomy OR LC) AND (ibuprofen OR "intravenous ibuprofen"); Cochrane: (laparoscopic cholecystectomy OR laparoscopic gallbladder surgery) AND (ibuprofen) AND (postoperative pain OR analgesia OR pain control) LC: laparoscopic cholecystectomy; NSAID: non-steroidal anti-inflammatory drugs

PICO component	Regimen	MeSH terms used	Keywords
Population (P)	Patients undergoing laparoscopic cholecystectomy	“Cholecystectomy, Laparoscopic”[MeSH]	Laparoscopic cholecystectomy; laparoscopic gallbladder surgery
Intervention (I)	Ibuprofen administration	“Ibuprofen”[MeSH]	Ibuprofen
Comparator (C)	Placebo or alternative analgesics (e.g., acetaminophen, dexketoprofen, pregabalin)	Not restricted in search	Placebo; acetaminophen; dexketoprofen; pregabalin
Outcome (O)	Post-operative pain control/analgesic efficacy	“Postoperative Pain”[MeSH]	Postoperative pain; pain control; analgesia
